# Anisotropic Silver Nanomaterials by Photochemical Reactions: Synthesis and Applications

**DOI:** 10.3390/nano11092226

**Published:** 2021-08-29

**Authors:** Vittorio Scardaci

**Affiliations:** Dipartimento di Scienze Chimiche, Università degli Studi di Catania, Viale A. Doria 6, 95125 Catania, Italy; vittorio.scardaci@unict.it

**Keywords:** silver nanoparticles, nanoplates, photochemistry

## Abstract

Silver-based nanoparticles have attracted a broad interest due to their outstanding optical and chemical properties and have been studied for applications in many fields. While different synthetic routes have been explored, photochemical synthesis has attracted a special interest for its limited use of chemicals and ease of control over the shape and size of the nanoparticles. This paper reviews the main factors affecting the synthesis of anisotropic silver nanoparticles, such as irradiation wavelength, pH, etc., and the role of specific key molecules, such as citrate. The paper is structured into different sections depending on how the synthesis is initiated; thus, after the introduction, the photochemical conversion reaction starting from nanoparticles, or seeds, obtained chemically, is covered, followed by reactions from nanoparticles obtained by laser ablation by seedless reactions. After that, the applications proposed for anisotropic nanoparticles obtained by the methods discussed in the previous sections are briefly covered and, finally, the conclusions and the author’s perspectives are given.

## 1. Introduction

Silver nanoparticles (Ag NPs) have been at the center of extensive research over the last two decades and more due to their outstanding chemical and optical properties, finding applications in a broad range of fields, including sensing, electronics, photonics and biology, to name a few [1,2,3,4,5]. The optical properties are related to a phenomenon known as surface plasmon resonance (SPR), consisting in the interaction of the conduction electrons of a metal NP with external electromagnetic fields [6]. The resulting SPR is then strongly dependent on geometrical intrinsic factors, such as size and shape of the NP, as well as external factors, such as the refractive index of the surrounding medium [6].

A number of different synthetic routes have been proposed over the years for the production of Ag NPs of different shapes [7]. For shapes other than spherical, most synthetic procedures involve two separate steps, as spherical NPs are first obtained, to be converted into a different shape in a second step. Typically, spherical Ag NPs, or seeds, can be synthesized through the chemical reduction of Ag^+^ by sodium boronhydride (NaBH_4_) in a water solution [8] and then transformed using other chemicals, such as hydrazine, in what is commonly known as seed-mediated growth [9,10,11]. Alternatively, Ag NPs can be initially produced by laser ablation in solution [12].

An alternative conversion method was proposed in the early 2000s by Jin et al., who developed a method to convert Ag seeds to anisotropic Ag NPs using only the action of light [13,14], thus avoiding the use of chemicals, such as those used for the chemical process, which present challenges in their handling and are harmful to the environment. The pioneering work carried out by Jin and co-workers opened up a new area of research focused on the photochemical conversion of Ag NPs into a variety of exotic shapes, both flat, such as discs, hexagons and triangles, and 3D, such as prisms, decahedra, rods, etc. Such area of research is the focus of this review, which aims to cover the synthetic procedures and analyze the conditions and factors leading to the control of the size and shapes of these anisotropic Ag NPs, as well as the mechanisms of reaction. Because conversion processes are quite different, depending on whether the Ag NPs are initially produced chemically or by laser ablation, these are divided into two separate sections (Section 2 and Section 3, respectively). Section 4, instead, covers the photochemical seedless synthesis, where the photoconversion is applied directly on a Ag salt without the step involving the formation of seeds. Finally, Section 5 provides a brief review of the applications proposed for photochemically produced Ag NPs, before the conclusions and perspectives are given in Section 5.

## 2. Photochemical Conversion of Chemically Produced Ag Seeds

**Figure 1:**
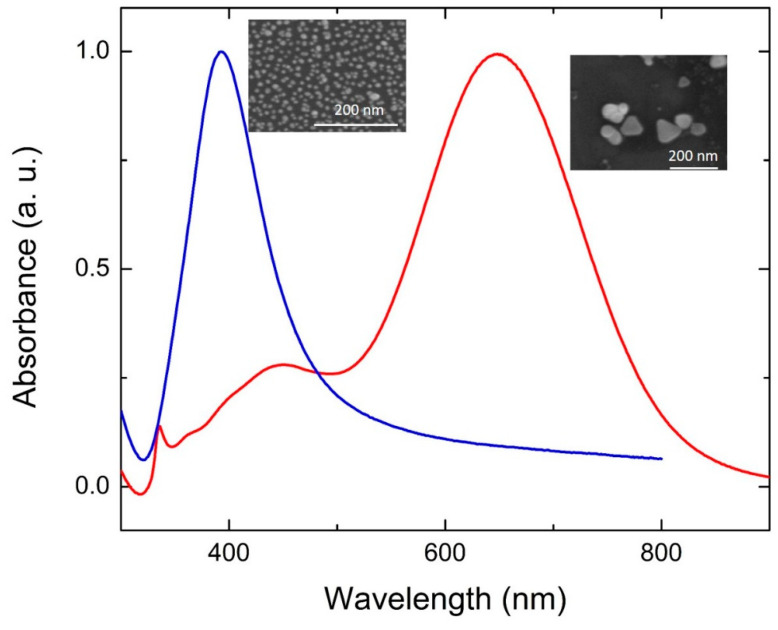
Examples of absorption spectra from spherical NPs (blue line) and flat NPTs (red line). Insets: SEM images from the corresponding structures.

**Figure 2:**
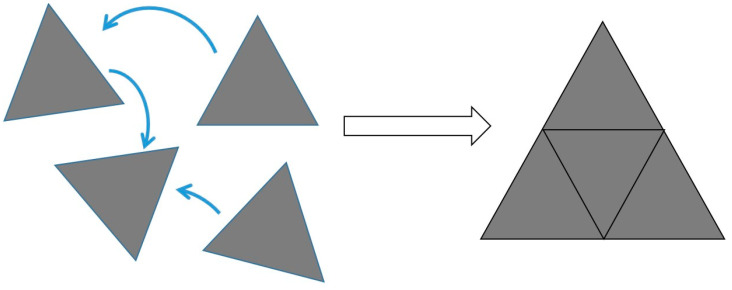
Schematic mechanism for the formation of larger nanoplates yielding a bimodal distribution in ref. [14].

This section covers the synthesis and mechanism of transformation of Ag seeds produced chemically and analyzes specific conditions for the reaction, such as irradiation wavelength, pH and Ag^+^ concentration. The shape change is typically characterized by scanning or transmission electron microscopies (SEM or TEM) and by simple UV-Vis absorption spectrophotometry. While the use of microscopies is straightforward, as they allow a direct observation of the NP shapes, absorption spectrophotometry is an indirect assessment. As an example, Figure 1 shows a comparison of absorption spectra from spherical NPs and flat nanoplates (NPTs). Significant differences can be observed. Spherical NPs show a single feature around 400 nm arising from an isotropic SPR. On the other hand, Ag NPTs show multiple features at different wavelengths. The first report of Ag NP photochemical shape conversion, as already mentioned, was published in 2001 by Jin et al. [13]. They observed that the initially yellow solution, typical of spherical NPs, turned green, then blue, over a period of 70 h under irradiation from a 40 W fluorescent light, with broad emission from 300 to 700 nm. The reaction environment also contained trisodium citrate (TSC) and Bis(p-sulfonatophenyl)phenylphosphine dihydrate dipotassium (BSPP). The absorption spectrum gradually evolved from the blue line, representing spherical NPs, to the red line, representing flat NPTs, in Figure 1. Here, the main SPR peak lies around 700 nm, suggesting a significant shape change that was confirmed by TEM imaging, showing a transformation from 8 nm spheres to triangular NPTs with an edge length of around 100 nm [13]. On the other hand, the reaction did not take place in darkness. TEM and electron energy loss spectroscopy (EELS) also highlighted that the NPT base facets have a (111) orientation. Through the aid of simulations, four different features can be identified in the NPT spectrum showed in Figure 1. The most intense is the in-plane dipole mode (650 nm in Figure 1). Its position depends on the size and aspect ratio of the NPTs and is also sensitive to the sharpness or truncation of the tips and can thus span most of the visible and the closest part of the near IR spectrum, according to different reports. A lower intensity feature, but not less important, is the out-of-plane quadrupole mode around 340 nm, which is a strong indication that the particle is anisotropic. Other features are the in-plane quadrupole and out-of-plane dipole modes in the 400–500 nm region, which are usually less visible and can easily overlap or be shadowed by residual spherical NPs [13]. The authors suggested a three-stage mechanism, in which the induction period yields smaller seeds by dissolution or fragmentation of larger NPs, then small NPTs as small as 5–10 nm begin their formation, finally acting as seeds for the formation of the final larger NPTs. Results from this first experiment were further refined by the same group, this time restricting the irradiation wavelength range using a 40 nm bandpass filter around 550 nm on a xenon lamp and using a secondary irradiation beam [14]. The authors observed a bi-modal distribution of NPT, centered at 70 nm and 150 nm, with an absorption spectrum showing two distinct in-plane dipole resonance peaks at 680 and 1065 nm and proposed a mechanism in which four triangles with size around 70 nm form a larger triangle with size close to 150 nm (Figure 2). This is supported by the spectral evolution that sees the arising of the 1065 nm SPR band delayed if compared to the 680 nm one. Interestingly, using a secondary beam at 450 nm, exciting the in-plane quadrupole mode, suppressed the formation of the 150 nm NPT and the corresponding 1065 nm band. This also happened with a secondary irradiation at 340 nm exciting the out-of-plane quadrupole [14]. Moreover, using a 340 nm secondary beam and tuning the primary irradiation beam, unimodal distributions could be achieved with size ranging from 35 to 120 nm and SPR bands from 500 to 900 nm. Such results then highlighted, for the first time, the importance of plasmon excitation in the photochemical conversion of Ag NPs [14], which was further investigated by many other groups in the following years. 

The following subsections analyze the fundamental factors that determine the mechanisms of growth of Ag NPs into different shapes under light irradiation.

### 2.1. Role of Citrate

The work carried out by Sun et al. is pivotal in understanding the photochemical conversion of Ag NPs, as they highlighted, for the first time, the key role of TSC [15]. Here, TSC was used along with polyvinyl pyrrolidone (PVP) in the seed conversion reaction and it was observed that, while PVP was needed to achieve a monodisperse distribution of seeds smaller than 10 nm, no shape conversion was achieved if TSC was not in the mixture. Moreover, if TSC was in solution from the seed synthesis stage, or was added afterwards, made no difference in the shape conversion [15]. Maillard et al. elaborated further on the role of TSC [16]. Using also silver nitrate (AgNO_3_) in the photoconversion reaction environment, they highlighted the fact that TSC acted as both a capping agent stabilizing the particles in the solution and a photoreducing agent for Ag^+^ ions through the reaction:

Citrate + λ → Acetone-1,3-dicarboxylate + CO_2_ + 2e^−^

While the two remaining carboxylic groups bound to the Ag NP surface, they transferred the electron to the NP allowing Ag^+^ reduction on the surface and, consequently, its growth. Notably, no conversion took place in the absence of TSC or Ag^+^ ions [16].

**Figure 3:**
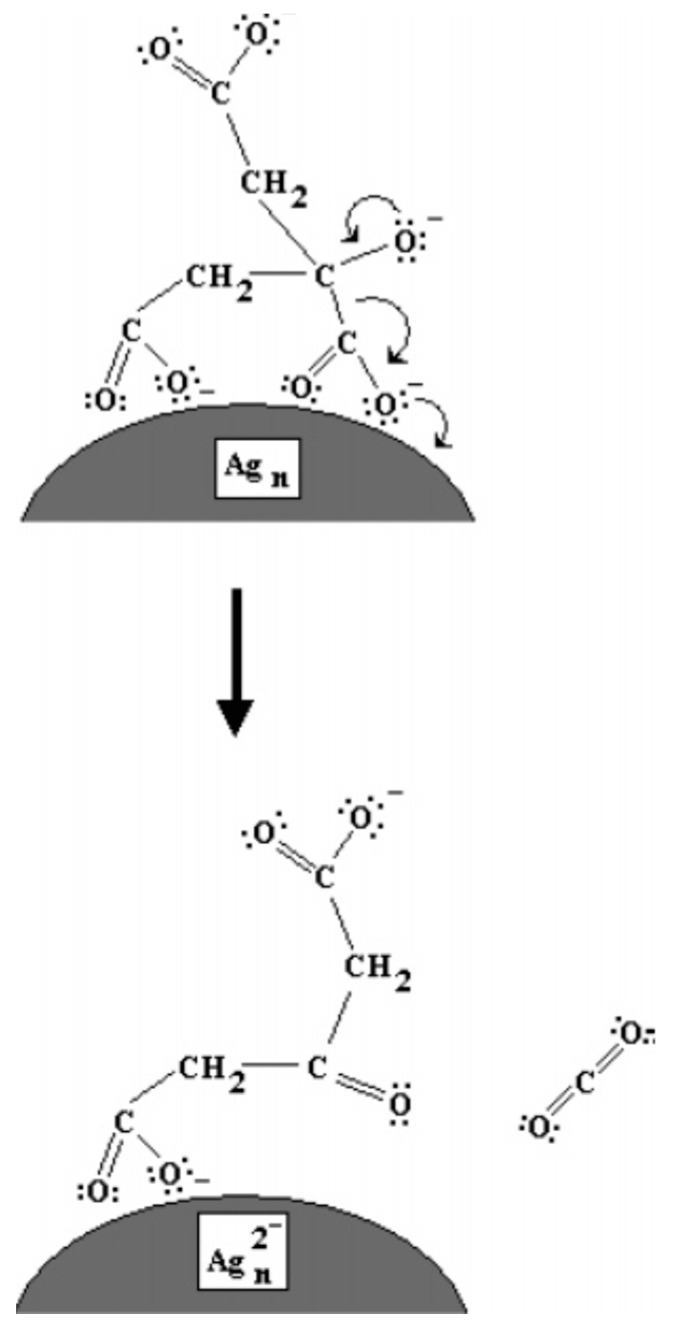
Mechanism proposed by Redmond et al. for the photoreduction of Ag NPs by photoxidation of citrate on their surface. Reprinted with permission from ref. [17]. Copyright 2007 American Chemical Society.

A few years later, Redmond et al. built on the work carried out by Maillard et al. to further elucidate the TSC role in the photoconversion of Ag NPs [17]. Here, the authors used Ag nanocrystals that were formed by thermal annealing of a Ag film deposited by evaporation on an ITO-covered glass slide [17]. When such system was immersed in the reaction environment proposed in ref. [16], containing TSC and AgNO_3_, under irradiation, a potential could be measured on the ITO electrode, supporting that TSC was oxidizing on the Ag surface. Interestingly, the potential variation was higher when AgNO_3_ was absent, suggesting that the charge from citrate is preferentially transferred to Ag^+^ ions [16]. The proposed mechanism is schematized in Figure 3. Jia et al. hypothesized that TSC might preferentially react with some crystal faces of silver [18,19]. Later, simulations performed by Kilin et al. suggested that TSC would preferentially bind to the (111) facets of silver [20], which have been shown to constitute the basal plane of Ag NPTs [13,21]. Thus, in summary, TSC would bind to the (111) surface of silver. Under light irradiation, TSC would transfer electrons directly onto the Ag surface, allowing Ag^+^, present in solution, to reduce on the Ag NP surface, where TSC was not bound, thus growing the particle in two directions only and forming an NPT. Wu et al. measured the evolution of the TSC concentration over time and found that, while its variation was initially limited (50 min), it decreased rapidly in parallel with the increase in the SPR dipole band (up to 250 min) and plateaued afterwards for a three-stage mechanism [22]. The authors identified 0.27 M as the minimum TSC concentration to achieve a complete monolayer on the seeds, while, below that concentration, the photoconversion progressed slower and, below 0.09 M, no photoconversion occurred [22].

It could be argued that the photochemical conversion was first demonstrated with no Ag^+^ ions in solution [13,14]. This was solved by Xue et al. [23], who suggested that the key is having a source of Ag^+^ in solution. If this is not provided by actively adding an Ag^+^ salt, it is the oxygen present in the solution that oxidizes Ag to Ag^+^. Indeed, they proved that, by carrying out the reaction under nitrogen, no photochemical conversion could be observed without added Ag^+^. Notably, the BSPP is needed to coordinate the Ag^+^ ion in solution and accelerate its dissolution [23]. The effect of TSC concentration, along with other factors, was studied by Kim et al. [24] in the synthesis of round-shaped NPTs (or nanodisks). They found that, under irradiation of a metal halide lamp, increasing TSC concentration lead to smaller NPTs, while decreasing NPT concentration provided larger NPT. This was also observed for NPTs grown from spherical NPs made by laser ablation [25]. The same applies to Ag^+^ concentration [24]. The authors were also able to tune the size of the round NPTs by controlling the dilution of the seed solution during irradiation, finding that higher dilution, thus lower seed concentration, lead to larger NPTs and vice versa, similarly to seed-mediated growth methods [9].

### 2.2. Influence of Irradiation Wavelength and Intensity

**Figure 4:**
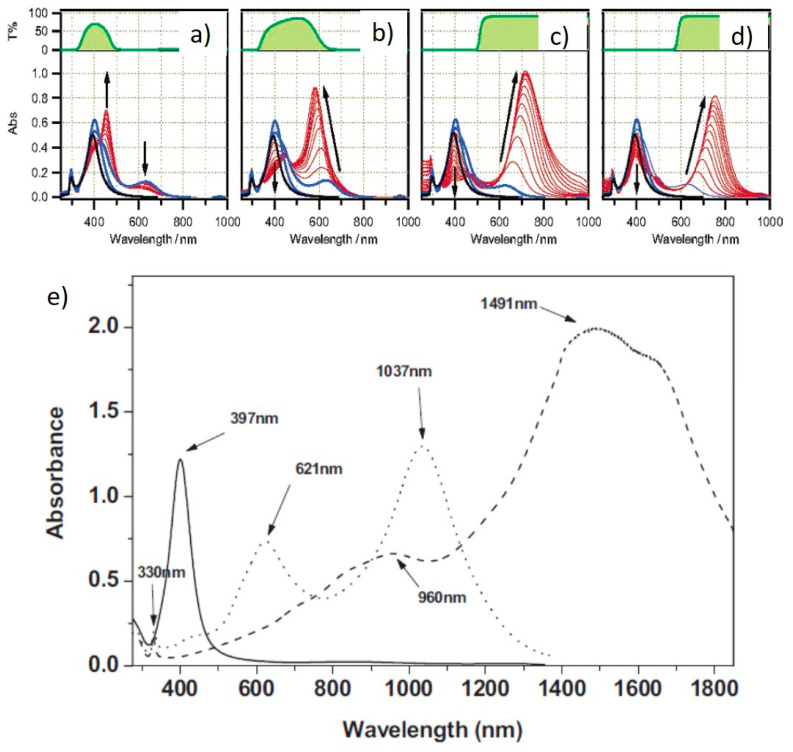
Absorption spectra of Ag NPs irradiated by different wavelengths [26,27]. (**a**) Narrow band color filter, (**b**) wide band color filter, (**c**) 515 nm band pass filter, (**d**) 590 nm band pass filter and (**e**) green (dotted line) and red (dashed line) LEDs, along with starting NPs (solid line). Adapted with permission from ref. [26], Copyright 2003 American Chemical Society (**a**–**d**). Reprinted permission from ref. [27], Copyright 2006 WILEY-VCH Verlag GmbH & Co. KGaA, Weinheim (**e**).

**Figure 5:**
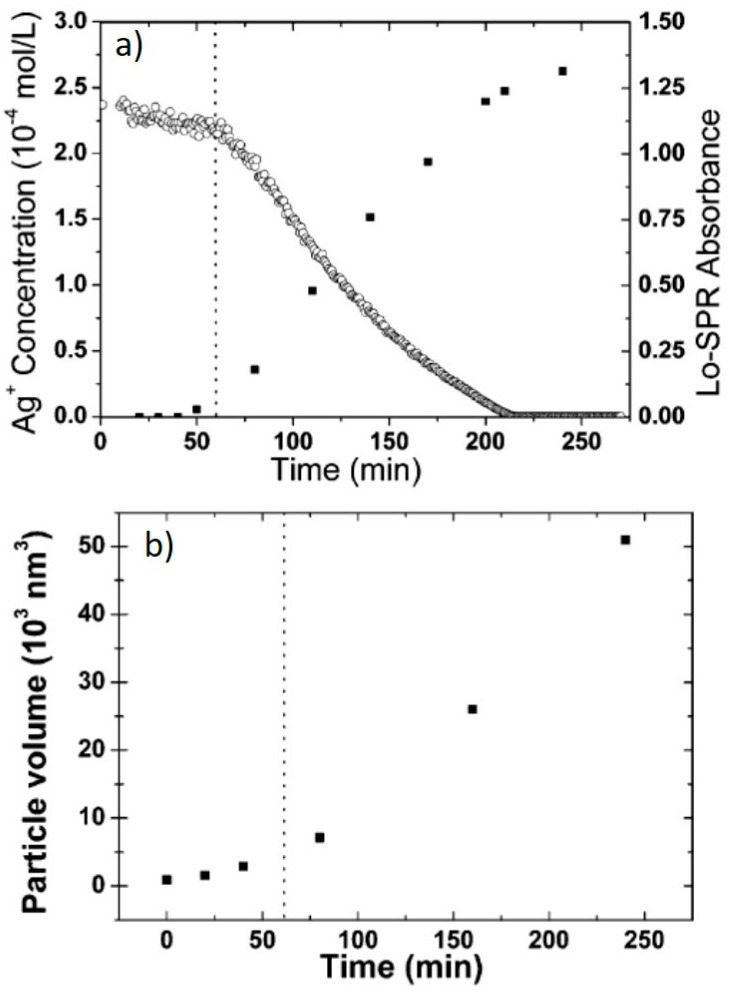
(**a**) Ag+ concentration evolution over time; (**b**) particle volume evolution over time, as estimated from TEM images. Adapted with permission from ref. [29]. Copyright 2007 American Chemical Society.

A number of different reports addressed the influence of the irradiation wavelengths, using selected laser lines or LEDs or applying color filters to broadband lamps. After the importance of SPR excitation was first highlighted [14], many groups investigated this matter further. Callegari et al. applied a range of color filers to a fluorescent tube light source for the photochemical conversion of Ag NPs [26]. The results are summarized in Figure 4a–d. By using a narrow bandpass filter (349–467 nm), only a few triangles were obtained, along with other small structures and aggregates and the absorption spectrum shows a prominent feature at 450 nm (Figure 4a), while, using a slightly broader filter (354–589 nm), small triangles were the majority, forming a more intense feature around 600 nm (Figure 4b). Using edge filters from 515 nm and 590 nm allowed the formation of larger triangles with the SPR around 700 nm (Figure 4c,d). Similarly to ref. [13], the authors identified three stages, with an induction period, a period in which some intermediate structure formed and the final growth period when the final triangular structures formed [26]. Bastys et al. made a little step further, using LEDs for irradiation in the green (518 nm) and red (653 nm) [27]. They observed the formation of bimodal distributions in both, as, by green irradiation, the SPR shifted to 621 nm and 1037 nm (110 nm size), while, by red irradiation, the SPR moved to 960 and 1491 nm, with average size of 110 nm and 242 nm, respectively, and similar thickness of 10 nm (Figure 4e) [27]. Similar results were obtained by Rocha et al., who obtained a 600 nm SPR under a 500–550 nm irradiation by color filter on a Xenon lamp and a 700 nm SPR under a 600–650 nm irradiation [28]. The same group further investigated the matter by deliberately choosing irradiation wavelengths far from that of initial seeds and by measuring the Ag^+^ concentration while the reaction occurred [29]. At a 600–650 nm excitation, during the initial 60 min of the reaction, the Ag^+^ concentration decreased very slowly; then, after 60 min, until 240 min, the decrease was much faster (Figure 5a). The change in Ag^+^ consumption rate is coincident with the formation and increase in intensity of an SPR band at 750 nm, indicating a two-step process in which the size of the seeds initially increased, with very few NPTs forming, followed by NPT formation. This is also supported by the particle volume evolution with time (Figure 5b) [29]. A similar experiment, performed using a 500–550 nm band-pass filter, showed that the first stage was almost indistinguishable, while, for 700–750 nm excitation, it lasted up to 320 min. It is possible that a longer wavelength is not energetic enough to efficiently activate the TSC photoreduction on the Ag NP surface, according to the mechanism described in the previous section; thus, the photochemical conversion reaction progresses at a much slower rate as the wavelength decreases. This was confirmed by Thrall et al., who performed photovoltage measurements at different wavelengths [30], observing photovoltage (and photocurrent) increase at lower wavelengths (i.e., higher photon energy) and suggesting a charge transfer mechanism from the Ag NP to the adsorbed TSC molecule that triggered the process mentioned in the previous subsection.

**Figure 6:**
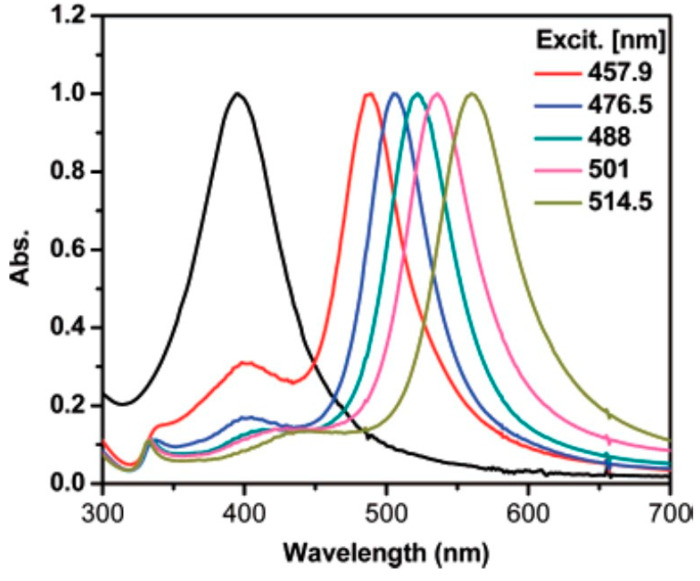
SPR bands from Ag NPTs irradiated by Ar-ion laser lines. Reprinted with permission from ref. [31]. Copyright 2007 American Chemical Society.

Zheng et al. made a step further and irradiated a seed solution using selected lines from an Ar-ion laser [31]. They observed that, by increasing the laser line wavelength, the SPR of the obtained NPT also red-shifted (Figure 6). While similar trends can also be observed elsewhere [32], by comparing the absorption spectra, it can be observed that irradiation by laser lines yielded narrower SPR bands than other irradiation sources. However, it is worth mentioning that expensive light sources, such as lasers or LEDs, are not strictly needed to achieve photoconversion of NPs into NPT. Indeed, Tang et al. achieved this result using a completely free source, such as sunlight, obtaining SPR bands between 500 and 700 nm [33]. 

**Figure 7:**
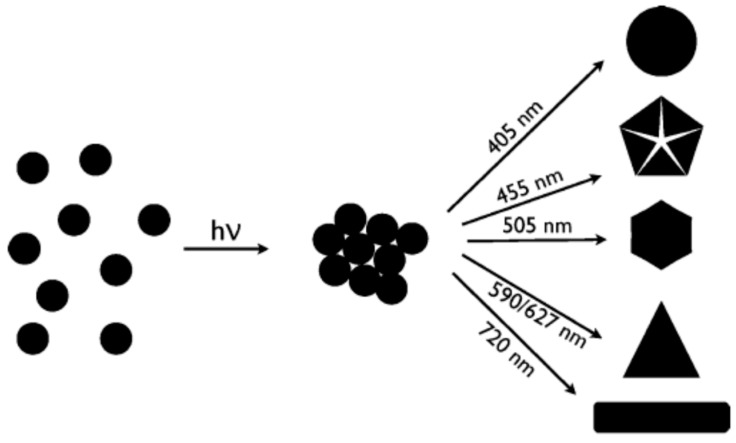
Different shapes obtained by irradiating very small Ag seeds by different wavelengths. Reprinted with permission from ref. [34]. Copyright (2010) American Chemical Society.

Another significant step further was achieved by Stamplecoskie et al., who managed to synthesize anisotropic NPs with different shapes by irradiating a Ag NP seed solution at different wavelengths by LEDs [34]. According to the authors, the critical step was obtaining very small seeds (3–4 nm), unlike previous reports, where seeds were 8–10 nm. While they did not speculate on the mechanism leading to different shapes, they could obtain discs, dodecahedra, hexagons, triangles and rods by varying the irradiation wavelength from 405 nm to 720 nm, as shown in Figure 7.

The effect of irradiation intensity was studied by Wu et al. under a 514 nm laser line [22]. No reaction took place and no spectral change was observed at intensity <2 mW/cm^2^, while, for values up to 10 mW/cm^2^, the yield was maximum. The photoconversion efficiency and rate decreased for intensity > 10 mW/cm^2^ [22].

Wang et al. investigated the effect of temperature on the morphology of the obtained NPs and found that, at 20 °C, the formation of nanodecahedra was predominant, while triangular nanoplates tended to form at 40 °C [35]. A mixture of shapes was obtained at 30 °C. The same group expanded the mentioned work coupling different irradiation wavelengths (from 478 nm to 590 nm) to a range of temperatures (from 5 °C to 40 °C) [36]. They observed a similar shape trend with temperatures, with the SPR that was red-shifted as the excitation was also red-shifted, in agreement with previous studies. Thus, overall, it was broadly observed that irradiation by visible light produced structures with SPR that red-shifted as the excitation re-shifted. However, pushing the irradiation wavelength towards the near IR made the photoconversion reaction much slower.

### 2.3. Effect of pH

The effect of pH was studied by Xue et al. on the basis that the Ag NP surface is coated by negatively charged particles, such as TSC, which must be sensitive to variations of pH [32]. They observed that, by irradiating a seed solution using a halogen lamp with a 550 nm band-pass filter at pH = 9.5, which is in the range of the natural pH of such systems if no other perturbation or buffering occurs, a bimodal distribution was obtained, with a double SPR band at 616 nm and 1032 nm. On the other hand, if the pH was increased to 11, only one SPR band at 616 nm was obtained, suggesting a unimodal distribution [32]. The authors highlighted that, while the negative charge on the surface should prevent fusion between particles, if light excited the dipole SPR, optical attractive forces arose that lead to the bimodal distribution. This could be prevented only increasing the pH, thus stabilizing the negative charge on the surface further. Moreover, no reaction occurred at pH > 12, as the high concentration of OH^−^ tended to form AgOH, subtracting it from the solution, similarly to what could be observed adding NaCl that formed AgCl [32]. Nguyen et al. found that the best conditions for photoconvesion were at pH = 9, without however providing an interpretation [37].

### 2.4. Other Shapes

**Figure 8:**
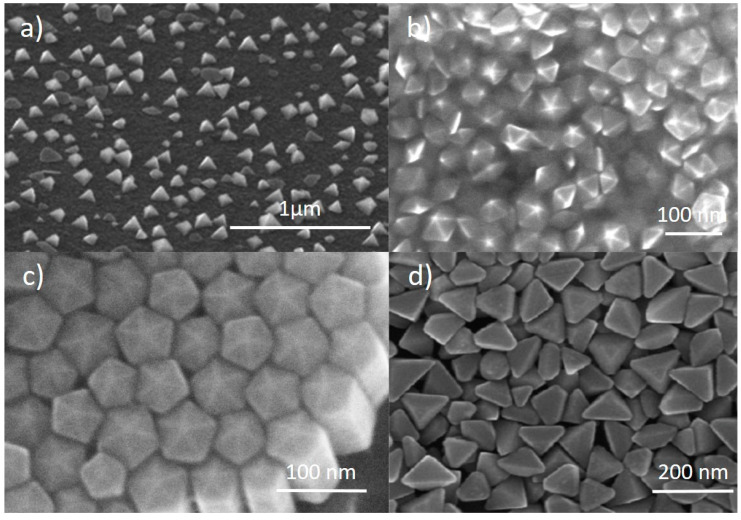
SEM images from: (**a**) Ag nano tetrahedra. Reprinted with permission from ref. [38]. Copyright (2008) American Chemical Society. (**b**) Ag nanodecahedra. Reprinted with permission from ref. [39]. Copyright (2008) American Chemical Society. (**c**) Ag pentagonal nanorods. Reprinted with permission from ref. [40]. Copyright (2009) American Chemical Society. (**d**) Ag nanobipyramids. Reprinted with permission from ref. [43]. Copyright 2010 American Chemical Society.

While most studies on the photochemical conversion of Ag NPs focus on flat nanoplates as the final result, there are some other studies that report the formation of more exotic 3D shapes [33,38,39,40,41,42,43,44]. The formation of tetrahedra (Figure 8a) was reported by Zhou et al. using two different capping agents, tartrate and TSC, during irradiation [38]. Specifically, a seed solution was irradiated for 9 h under a sodium lamp in presence of tartrate and subsequently irradiated for 20 h in presence of TSC. During the first period, the SPR of the seeds blue-shifted from 404 nm to 395 nm, suggesting some sort of rearrangement from the seeds, promoted by tartrate. Afterwards, a new feature at 642 nm was formed upon TSC addition, yielding the final tetrahedra [45].

The formation of decahedra (Figure 8b) was first reported by Pietrobon et al., who used L-arginine in the growth solution as a photochemical promoter [39], while NPTs were formed, similarly to many other reports already discussed, if no L-arginine was present. A series of regrowth stages could be performed to enlarge the size of the obtained structures, which acted as seeds, or precursors, for further regrowth. In this case, the authors could control the size of the decahedra from 40 nm to 120 nm [39]. Building on this method, Lu et al. could also control the size and SPR of nanodecahedra by combining different irradiation wavelength, seed concentration and precursor concentration for regrowth, obtaining structures with an SPR from 490 nm to 590 nm [44]. Zheng et al. reported the formation of decahedra with an SPR close to 500 nm by irradiating a seed solution using a 465 nm LED [41]. The authors suggested that carrying out the reaction in a cooling bath promoted the formation of decahedra, while a mixture of shapes was formed if no cooling was applied. Tang et al. obtained nanodecahedra using UV light irradiation of a Ag seed solution [33]. Nanodecahedra of 35–45 nm in size were used by Pietrobon et al. as precursors for the growth of pentagonal faceted nanorods (Figure 8c) [40]. The growth was obtained at 95 °C adding AgNO_3_ to the solution. By adding different amounts of AgNO_3_, different lengths could be obtained, with aspect ratios up to 12 and SPRs from 500 to 850 nm. The synthesis of nanodecahedra was also studied by Ye et al., who investigated the effect of temperature [46]. They started from AgNO_3_, TSC and a photoinitiator (I-2959) and irradiated by UV (355 nm) first, to obtain the seeds, and with visible light (455–465 nm LED) afterwards, to drive the photochemical conversion, which ended up with nanodecahedra in the range of 30–40 nm. By varying the temperature between 30 and 70 °C, the authors observed larger SPR red-shifts at lower temperature, as well as overall faster reactions at higher temperatures [46].

Triangular bipyramids (Figure 8d) were produced by Zhang et al. irradiating a solution containing AgNO_3_, TSC, BSPP and NaOH using a halogen lamp [42,43]. They demonstrated that, using a range of band-pass filters from 500 nm to 650 nm, the SPR of such structures could be tuned from 530 nm to 770 nm, with size ranging between 100 and 200 nm [42]. Moreover, it was found that the optimal reaction conditions to obtain the bipyramids were at a pH in the 10–11 range and BSPP/Ag^+^ concentration ratio around 1, where the reaction was also faster [43]. Decreasing the pH to 9 and increasing the BSPP/Ag^+^ concentration ratio to >2 caused the formation of truncated bipyramids, while further decreasing the pH yielded NPTs [43].

### 2.5. Interconversion between Shapes

A few reports have been published reporting the interconversion between Ag NPs of different shapes [47,48,49,50]. Tang et al. showed that continuing irradiation under a sodium lamp for up to 400 min could transform the initial triangular NPTs, obtained by the already described method with TSC, into nanodiscs [47]. By adding further TSC, the so-obtained nanodiscs turned again into triangular NPTs. Because the final nanoplates were larger than initial ones, this could be seen as a unique growth process and was not reversible. Zhang et al. reported that, under UV irradiation, triangular NPTs could be converted into nanodiscs [48]. The process involved the progressive etching of the tips of the triangles until they became completely round in shape and was reflected by the absorption spectrum, which showed a continuous blue-shift of the in-plane dipole SPR [48].

**Figure 9:**
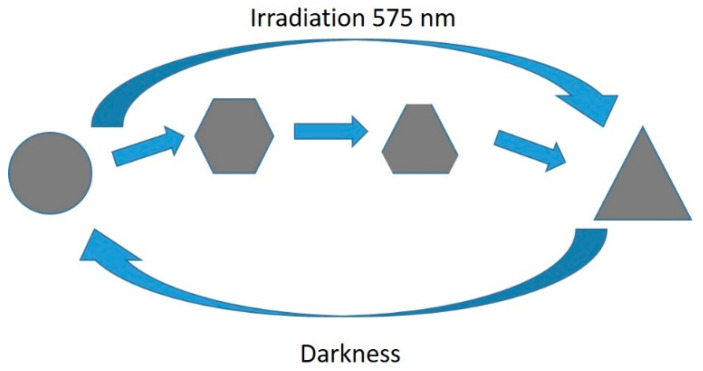
Interconvesion mechanism between round and triangular NPTs [49,50].

A reversible interconversion between triangular and round NPTs was reported by Lee et al. [49]. The authors reported that triangular NPTs, obtained by a standard photoconversion method with TSC, could be transformed into nanodiscs over a period of 24 h in darkness if BSPP was added to the solution. Such nanodiscs could be converted back to triangles if irradiated for 20 min at 575 nm [49]. This effect was attributed to the BSPP etching the more reactive triangle tips when TSC did not react. While the authors demonstrated the process of cyclability up to eleven times, BSPP was irreversibly consumed during the process; the recovery was not complete, as can be observed by absorption spectra in ref. [49]. This was restored by adding fresh BSPP [49]. This conversion could be further controlled by irradiating for shorter intervals of time, as by doing so hexagons and truncated triangles could also be isolated from the reaction environment [50]. This overall process is schematized in Figure 9.

## 3. Photochemical Conversion of Laser Ablated Silver Nanoparticles

Laser ablation in liquid (LAL) is a versatile tool for the production of a wide range of metal, as well as non-metal, NPs, consisting in the focusing of a high-power pulsed laser onto a target immersed in a liquid [12]. By this method, NPs can be produced in a liquid without any other chemical. The investigation of the photoconversion of Ag NPs obtained by LAL began in parallel with studies on seed-based photoconversion, as the first report appeared in 2003 published by Tsuji et al. [51]. Here, after NPs were produced by LAL using a 1064 Nd:YAG laser, these were irradiated by a 355 nm laser with no further chemical added, yielding the formation of tubular structures, the absorption spectrum of which was narrower than that of the initial NPs but still centered around 400 nm, with no quadrupole features [51]. Later, they studied the effect of laser fluence on the morphology of the obtained structures and found that, for fluences of 50–100 mJ/cm^2^, triangular NPTs formed, while fluences >150 mJ/cm^2^ yielded a wide mix of structures, including wires, spheres and various fragments [52]. If halides were added to such a system before irradiation, the yield in NPT significantly increased, with iodide more efficient than chloride [53,54]. The authors suggested a mechanism in which the halide formed a complex with Ag that, in turn, was transformed to NPTs by irradiation [54]:

Ag^+^ + 2X^−^ → AgX^−^ → NPT

Adding PVP to the solution at the irradiation stage, at concentration of up to 6 mM, increased the formation of nanocrystals in solution, along with a decrease in size [55,56]. While a mixture of shapes seemed to be formed by TEM analysis, spectral observations did not show any quadrupole feature, probably because the yield was low compared to the initial spherical particles that were still visible. PVP concentrations of 1 mM or lower did not allow the formation of any nanocrystals [55,56].

**Figure 10:**
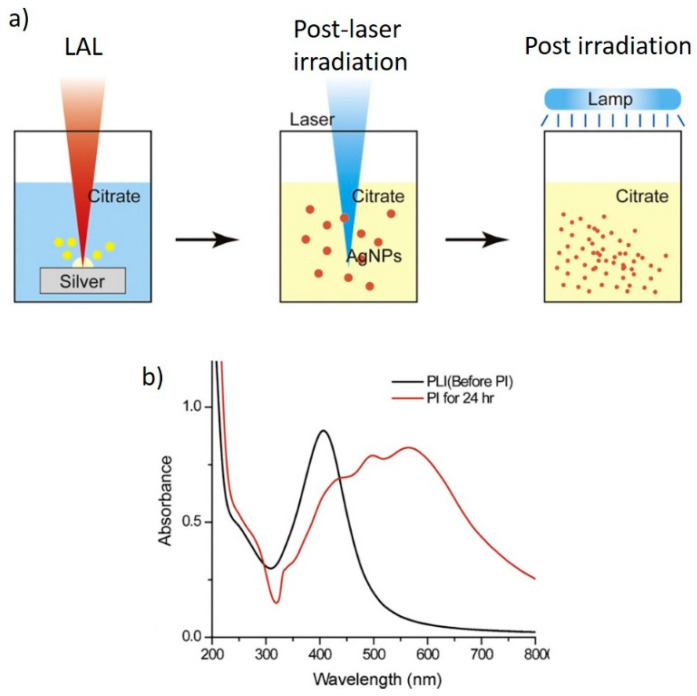
(**a**) Scheme of the LAL-based process allowing the formation of NPTs; (**b**) absorption spectra from NPs after the post laser irradiation (PLI) and post irradiation (PI). Reprinted/adapted with permission from ref. [57]. Copyright 2011 Elsevier.

Only when TSC was added to the reaction environment, a clear indication of the formation of NPT could be obtained both by electron microscopy imaging and by optical absorption spectroscopy [57]. A detailed investigation, again by Tsuji et al., evidenced that, by adding TSC after LAL and performing a post-irradiation by a Xenon lamp (thus not a post-laser irradiation), the outcome did not change significantly from previous reports, as 20 nm spherical particles and very few nanocrystals were formed. Only when a post irradiation followed a post-laser irradiation, the formation of NPTs could be observed both by electron microscopy and by spectroscopy (Figure 10a,b). The authors identified, again, three stages during the post irradiation process (i.e., after the post-laser irradiation). Initially, the spherical NPs reduced in size to roughly 5 nm (up to 2 h); then, triangular NPTs were observed (up to 13 h). Finally, other non-spherical nanostructures were formed (up to 24 h). Thus, to obtain triangular NPTs, the post irradiation process should not exceed 13 h [57]. A faster process was adopted by Condorelli et al., who did not perform a post-laser irradiation step but only irradiated the solution by a white LED lamp for 6 h [58]. Here, the authors, again, used TSC from the beginning and added H_2_O_2_ during the irradiation step. H_2_O_2_ was expected to both reduce and oxidize Ag, according to the reactions

2Ag + H_2_O_2_ → 2Ag^+^ + 2OH^−^

**Figure 11:**
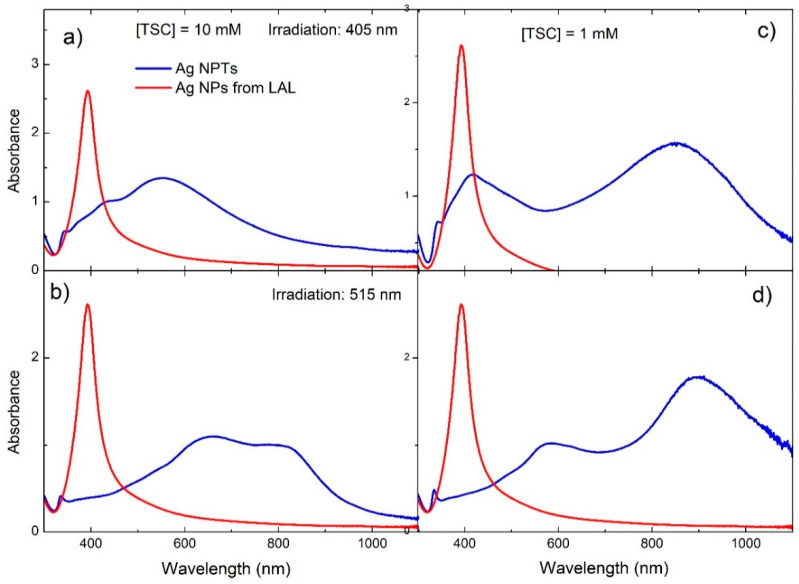
Absorption spectra from Ag NPTs produced by LAL followed by LED monochromatic irradiation (blue lines) and Ag NPs from LAL (red lines). (**a**,**b**) TSC concentration, 10 mM; (**c**,**d**) TSC concentration, 1 mM; (**a**,**c**) irradiation at 405 nm; (**b**,**d**) irradiation at 515 nm. Adapted from ref. [25]. Copyright 2020 American Chemical Society.

H_2_O_2_ + Ag^+^ + 2OH^−^ → 2Ag + 2H_2_O + O_2_

and worked with the TSC to convert spherical NPs to triangular NPTs [58]. A similar process was used from the same group to study the effect of TSC concentration and irradiation wavelength [25]. It was found that higher TSC concentration (10 mM) produced smaller NPTs (80–100 nm) with a dipole SPR mainly in the visible (600–850 nm), while lower TSC concentration (1 mM) allowed the formation of larger NPTs (120–150 nm) with a dipole SPR in the near IR (750–1000 nm), similarly to ref. [24]. It was also found that, under 730 nm irradiation, like in previous reports, the yield of NPTs was very lower than other wavelengths at any TSC concentration, while 515 nm excitation seemed to produce a bimodal distribution, as can be inferred by the absorption spectra in Figure 11.

A photoconversion process of Ag NPs prepared by LAL (532 nm ablation wavelength) was also studied by Verma et al., who investigated the effect of TSC concentration and pH [59]. They found that the yield of Ag NPs after LAL was highest at a 10 mM TSC concentration and pH between 7 and 11 (they did not show data for pH > 11). Upon irradiation under white light, the optimal TSC concentration was still 10 mM and pH = 10, while, when slightly increasing to 25 mM, no NPTs could be formed. At such conditions, the introduction of Ag ions (0.1 mM) further increased the yield of the process [59].

## 4. Seedless Photochemical Conversion

While, in the previous sections, we described photochemical conversion processes for Ag NPs that involved the initial formation of spherical particles, either by chemical reaction or by LAL, this section describes photochemical reactions that start directly from Ag^+^ salts in solution. The first of such reports appeared in 2006 from Tian et al., who irradiated a solution containing AgNO_3_, TSC and NaOH for 3 h using a tungsten lamp and obtained triangular NPTs of 150 nm average size and SPR at 1052 nm [60].

**Figure 12:**
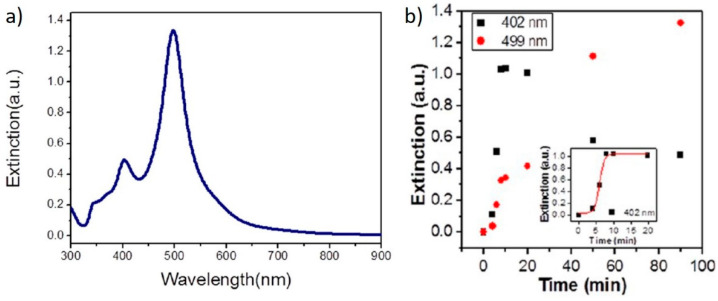
(**a**) optical absorption spectrum from Ag nanodecahedra produced by seedless photochemical growth; (**b**) time evolution of the SPR bands of Ag nanodecahedra during the growth reaction. Reprinted with permission from ref. [61]. Copyright 2012 American Chemical Society.

A few years later, Yang et al. irradiated, for 90 min, a solution containing AgNO_3_ and TSC with a set of 465 nm LEDs and obtained monodisperse nanodecahedra, showing a narrow feature around 500 nm (Figure 12a) [61]. The authors speculated that the reduction of Ag^+^ by TSC in solution was quite slow; nevertheless, some Ag seeds sis form and, once formed, they allowed the formation of the nanodecahedra by plasmon excitation. This proposed mechanism is supported by following the reaction over time by absorption spectroscopy, where they observed a feature at 402 nm, attributable to spherical particles, first increasing its intensity, then decreasing, with the formation of a band at 499 nm attributed to the nanodecahedra (Figure 12b). Similar results were obtained by Tang et al., who used simulated sunlight to irradiate a solution of AgNO_3_ and TSC for 12 h [33].

Lu et al. reported the formation of anisotropic structures (NPTs and nanodecahedra) starting from a solution containing AgNO_3_, PVP and a photoinitiator (I-2959), which reduced Ag^+^ in solution by radical reaction under irradiation by a mercury lamp at 365 nm [62]. While the structures could be observed by TEM, no quadrupole feature could be observed by spectral examination. 

## 5. Applications of Anisotropic Ag NPs Synthetized by Photochemical Conversion

Anisotropic Ag NPs obtained by photochemical conversion have been investigated for a restricted range of applications, the most popular being surface enhanced raman spectroscopy (SERS). Table 1 shows a summary of the results published so far, to the author’s knowledge, including the type of nanostructure, the sample molecule and the enhancement factor, where available. Such structures show enhancement factors in the range 10^4^–10^7^; thus, more research and development efforts are needed to achieve competitiveness against other materials that show enhancement factors higher that 10^10^ or even 10^12^.

Another field of application that has been investigated for anisotropic Ag NPs is sensing based on the variation of the SPR at different refractive indices of the surrounding medium. The reference parameter here is the plasmon sensitivity, *S*, defined as the ratio between the wavelength shift and the refractive index change: *S* = *Δλ*/*Δn*. Condorelli et al. investigated the plasmon sensitivity of triangular NPTs by dispersing small aliquots of NPT solution in sucrose solutions at set concentrations, providing different refractive indices [58]. They reported a value of 374 nm/RIU (refractive index unit). This compares with 125 nm/RIU, recorded for spherical NPs, suggesting that the change in shape from spherical to triangular allows a significant enhancement of *S*. Later, a value of 460 nm/RIU was reported for triangular Ag NPTs obtained by irradiation at 730 nm, compared to ~330 nm/RIU under irradiation at 405 nm and 515 nm [25]. Such results are in line with those reported for Ag NPTs obtained by other methods [9,63,64]. While this property has been demonstrated by different authors in solution, a real sensing application has not been demonstrated. A step forward has recently been made, where plasmon sensitivity has been demonstrated on Ag NPTs deposited onto reusable PMMA cuvette walls, thus applicable to different liquids [65].

## 6. Conclusions and Perspectives

This paper reviewed the mechanism of synthesis of anisotropic Ag NPs by photochemical conversion and their applications. Section 2 covered the synthesis, mechanisms and the range of conditions necessary to develop Ag NPTs from chemically prepared NPs, or seeds. The key role of TSC in the synthesis was discussed and the effects of irradiation wavelengths and pH have been analyzed. Moreover, Section 2 also described the synthesis of 3D anisotropic shapes, such as nanodecahedra and the mechanisms of conversion between different non-spherical shapes. Section 3 described the photochemical conversion processes that have been implemented from NPs produced by LAL. Section 4 discussed the photochemical synthesis that have been proposed with no seed formation, where the light irradiation is applied directly on the Ag^+^ salt solution. Section 5, finally, covered the applications that have been so far proposed for anisotropic Ag NPs obtained by photochemical conversion, which include SERS and plasmonic sensing.

From a synthesis perspective, while a lot has been studied and many aspects have been clarified, it was suggested that TSC is not the only molecule that can serve its scope, but, rather, many di- and tricarboxylate molecules can perform the same role [66]. However, to the author’s knowledge, no such molecules, other than TSC, have been investigated, leaving an open question on how such a system would behave.

From an application standpoint, despite the good control over shape and size that many photosynthetic routes allow, this has been rather limited to SERS and plasmonic sensing. Furthermore, SERS experiments, as already mentioned, reported enhancement factors that are not competitive enough, while a real-life application of plasmonic sensing has not been demonstrated. Other applications, such as photocatalysis, photonics, wider sensing, biosensing and biology, are yet to be explored using the photochemical conversion. Applications demonstrated for Ag NPs, such as, for example, water desalination [67,68], may be attempted for structures with different shapes exploiting their anisotropy.

## Figures and Tables

**Table 1:** Summary of SERS reports from photochemically synthesized Ag NPs.

Nanostructure	Sample Molecule	Enhancement Factor	Author and Ref.
Nanoplates	1,4-bis[2-(4-pyridyl)ethenyl]-benzene	10^6^	Jia [19]
Nanoplates	Rhodamine 6G	10^4^	Condorelli [58]
Tetrahedra	Benzenethiol	10^7^	Zhou [38]
Pentagonal nanorods	Thiosalicilic acid	n.a.	Pietrobon [40]
Nanodecahedra	Ag nanodecahedra	10^6^	Lu [44]
Nanodecahedra	Rhodamine 6G	n.a.	Wang [35]
Nanodecahedra	Rhodamine 6G	n.a.	Yang [61]
